# Hydrothermal Ageing and Its Effect on Fracture Load of Zirconia Dental Implants

**DOI:** 10.3390/ma14113103

**Published:** 2021-06-05

**Authors:** Laurent Gremillard, Agnès Mattlet, Alexandre Mathevon, Damien Fabrègue, Bruno Zberg, Marc Stephan

**Affiliations:** 1 Univ Lyon, CNRS, INSA Lyon, UCBL, MATEIS, UMR5510, 69621 Villeurbanne, France; agnes.mattlet@u-bourgogne.fr (A.M.); alexandre.mathevon@insa-lyon.fr (A.M.); damien.fabregue@insa-lyon.fr (D.F.); 2 Straumann AG, PeterMerian Weg 12, 4052 Basel, Switzerland; bruno.zberg@straumann.com (B.Z.); marc.stephan@straumann.com (M.S.)

**Keywords:** zirconia, dental implant, hydrothermal ageing, strength

## Abstract

Due to growing demand for metal-free dental restorations, dental ceramics, especially dental zirconia, represent an increasing share of the dental implants market. They may offer mechanical performances of the same range as titanium ones. However, their use is still restricted by a lack of confidence in their durability and, in particular, in their ability to resist hydrothermal ageing. In the present study, the ageing kinetics of commercial zirconia dental implants are characterized by X-ray diffraction after accelerated ageing in an autoclave at different temperatures, enabling their extrapolation to body temperature. Measurements of the fracture loads show no effect of hydrothermal ageing even after ageing treatments simulated a 90-year implantation.

## 1. Introduction

Zirconia ceramics used in dental implants are mostly 3Y-TZPs, standing for tetragonal zirconia polycrystal, stabilized in the tetragonal phase by 3 mol.% Y_2_O_3_. This ceramic can exist at room temperature in two main phases: the stable monoclinic phase and the metastable tetragonal phase. It is important to preserve the metastable tetragonal zirconia phase after processing since this phase offers the highest mechanical properties (fracture strength up to 1.5 GPa as an order of magnitude). However, the tetragonal phase is prone to a tetragonal-to-monoclinic transformation in the presence of water. The martensitic tetragonal-to-monoclinic (t–m) phase transformation results in a 5–7% volume increase in the transformed grains [1]. In the presence of water, the t–m transformation proceeds from the surface to the bulk, accompanied by roughening and microcracking, and is called hydrothermal ageing or low temperature degradation (LTD).

The effects of ageing on mechanical properties can be diverse. Indeed, the most expected effect would be a strength decrease due to the microcracked surface. This was indeed observed in some materials [2]. The strength decrease can even reach a complete loss of integrity, as summarized in the reviews by Piconi [3] and Ramesh (Table 7 of ref [4]). The same microcracking may lead to delamination of the aged surface layer [5]. However, the volume increase associated with the t–m transformation may also induce compressive stresses around the transformed zone that may increase the measured strength [3,4,6,7]. Whatever the stress state of the near-surface area, both elastic modulus and hardness of the aged surfaces may be decreased [8,9], which is due to the microcracking more than to the presence of the monoclinic phase itself [10].

At room or body temperature, hydrothermal ageing is a rather slow phenomenon. To be conveniently observable, zirconia ageing kinetics need to be measured at high temperatures (typically above 100 °C). It thus becomes necessary to establish a correspondence between the ageing kinetics measured at high temperature and the ones expected at body temperature. Zirconia ageing kinetics may be described by fitting the evolution of the monoclinic fraction (*f*) with time (*t*) by a Mehl–Avrami–Johnson (MAJ) Equation:

(1) $$f=f_{min}+\left(f_{max}2212\;f_{min}\right)\times \left(1-\exp\left(-{\left(bt\right)}^{n}\right)\right)$$

where *f_max_* is the saturation monoclinic volume fraction, *f_min_* is the initial monoclinic volume fraction, and *n* is a constant (the “MAJ exponent”). *b* is a thermally activated parameter; in the 37–145 °C temperature range it follows an Arrhenius law:

(2) $$b=b_{0}\times \exp\left(-\frac{Q}{RT}\right)$$

where *b*_0_ is a constant (pre-exponential term), *R* is the gas constant, *Q* is the apparent activation energy, and *T* is the absolute temperature [11].

Knowing the activation energy *Q* enables one to establish a time–temperature equivalence for the tetragonal-to-monoclinic transformation due to hydrothermal ageing. As shown in Equation (3) (derived from Equations (1) and (2)), the same monoclinic fraction is reached at the (*t*_1_, *T*_1_) and (*t*_2_, *T*_2_) (time, temperature) couples if

(3) $$\frac{t_{1}}{t_{2}}=\exp\left(\frac{Q}{R}\left(\frac{1}{T_{1}}-\frac{1}{T_{2}}\right)\right)$$

This is particularly useful to establish predictive ageing kinetics at body temperature from high temperature data (between 70 and 150 °C). However, although numerous works have measured zirconia hydrothermal ageing kinetics, only few discuss the activation energy. Even the most recent reviews neglect this parameter, even though it has been shown that it may vary greatly from one material to another, depending not only on the starting powder, but also on the sintering schedule (for example, values from 41 to 115 kJ·mol^−1^ were measured on samples made from the same powder) [12]. In most cases, activation energies for hydrothermal ageing lie between 100 and 120 kJ·mol^−1^, resulting in a correspondence of 1 h in an autoclave at 134 °C for 1 to 4 years in vivo at 37 °C.

Therefore, assessing the durability of zirconia dental implants related to hydrothermal ageing relies on several steps. First, the ageing kinetics have to be measured at several temperatures so that the constants related to the MAJ Equation, in particular the activation energy, can be deduced, to enable extrapolation of the ageing kinetics to body temperature (37 °C). Second, in order to ensure that no fracture of implant can occur due to regular ageing, an assessment of the mechanical properties (mainly strength) has to be conducted before and after ageing (for ageing times corresponding to relevant durations in vivo). Third, it is interesting to assess the evolution of surface properties in order to ensure that no delamination can occur, in particular in the parts of the implants in contact with bone.

In the present work, this procedure was applied to dental one-piece zirconia implants (Straumann^®^ PURE Ceramic Implant, Institut Straumann AG, Basel, Switzerland). More specifically, the constants related to their ageing kinetics according to the MAJ Equation (*Q*, *b*_0_, and *n*) were determined in order to deduce their ageing kinetics at body temperature from accelerated ageing kinetics in the laboratory. Then, their load to fracture after ageing in conditions equivalent to up to 90 years at 37 °C were measured. Finally, scratch tests were conducted to detect potential surface degradation.

The results show that the activation energy of these implants is in the usual range (around 100 kJ·mol^−1^). No negative influence of hydrothermal ageing on the load to fracture of these zirconia implants nor on their surface mechanical properties were detected.

## 2. Materials and Methods

### 2.1. Microstructural Observations

Fractographic observations were conducted on fracture surfaces of as-received and aged samples using a scanning electron microscope (Supra 55VP, Carl Zeiss AG, Oberkochen, Germany) without coating the samples. Imaging was performed at a low acceleration voltage (1.5 or 2 kV) with an Everhart-Thornley detector for secondary electrons. Chemical composition was assessed by Energy Dispersive X-ray Spectroscopy (EDS) analyses, performed at 25 kV acceleration voltage in partial vacuum mode (20 torr), using an Oxford SDD spectrometer with Aztec software (Oxford Instruments, High Wycombe, UK).

### 2.2. Measurement of Ageing Kinetics

The ageing kinetics of 20 dental two-piece zirconia implants (10 mm length, 4.1 mm diameter) (Straumann^®^ PURE Ceramic Implant, Institut Straumann AG, Basel, Switzerland) were evaluated at 5 different temperatures (70, 110, 134, 145 °C) and for different durations of up to 4657 h. The equipment used for ageing was chosen according to the necessary temperature and times (indeed, it was not possible to conduct long experiments inside autoclaves, due to limits in their programming capacities and availability). For ageing at 110, 134, and 145 °C, the samples were placed in open water vials inside an autoclave (SanoClav LA-MCS, Wolf, Geislingen/Steige, Germany). For ageing at 95 °C, the samples were placed in a closed water vial inside a thermo-regulated oven (VT5050EK, Heraeus, Hanau, Germany). For ageing at 70 °C, the samples were placed in glass dishes filled with deionized water inside a thermo-regulated water bath (Polytest 20, Fisher Scientific, Illkirch, France). In all cases, the samples were in contact with deionized water during the ageing process, so that all experiments were conducted in the same conditions with regard to contact with water. The temperature sensors of the equipment were checked before ageing.

The implants were periodically taken out, cooled, and dried so that their monoclinic fraction could be analyzed by X Ray Diffraction (XRD). The fraction of the monoclinic phase on the surface of the implants was measured by XRD, in Bragg–Brentano configuration with Cu K_α_ radiation in a θ–2θ mode (2 θ ∈ [26–33°]), with a scan speed of 0.5°/min and step size of 0.05° (Geigerflex diffractometer, Rigaku, Tokyo, Japan). The samples were positioned vertically in the diffractometer, so that the whole length of the implant was analyzed at once.

The monoclinic content was then calculated using Garvie and Nicholson’s Equation [13]:

(4) $$X_{m}=\frac{I_{m\left(\bar{1}11\right)}+I_{m\left(111\right)}}{I_{m\left(\bar{1}11\right)}+I_{m\left(111\right)}+I_{t\left(101\right)}}$$

where *X_m_* is the integrated intensity ratio, *I_m(hkl)_* is the area of the (h k l) peak of the monoclinic phase, and *I_t(hkl)_* is the area of the (h k l) peak of the tetragonal phase (peak intensities were measured using the EVA software (Bruker AXS, Karlsruhe, Germany)). The experimental volume fraction (*f*) of the monoclinic phase (average over the penetration depth of the X-rays: for CuK_α_ radiations, 90% of the signal comes from the first 5 µm below the surface) was then determined by Toraya’s Equation [14]:

(5) $$f=\frac{1.311\times X_{m}}{1+0.311\times X_{m}}$$

### 2.3. Assessment of Fracture Loads

Eighty dental one-piece zirconia implants (14 mm length, 3.3 mm diameter) (Straumann^®^ PURE Ceramic Implant, Institut Straumann AG, Basel, Switzerland) and 20 titanium implants (14 mm length, 3.3 mm diameter) (Standard Plus Implants, Institut Straumann AG, Basel, Switzerland) were supplied by Straumann for the assessment of their fracture load. Before fracture tests, some samples were aged in an autoclave at 134 °C for different durations to mimic in vivo ageing duration of 22.5, 45, and 90 years at 37 °C.

Before mechanical testing, the samples were embedded in 1 cm diameter × 1 cm height cylinders made of epoxy resin with a Young’s modulus of 5.8 GPa (RenCast CW 5156–1/Ren HY 5158, hardened at 55 °C during 15.5 h) (Huntsman Advanced Materials, Basel, Switzerland). The embedding depth was determined to ensure that the geometrical requirements of the ISO 14801 standard [15] were met.

The fracture tests were carried out on an Instron 8872 universal testing machine (Instron, Elancourt, France) using a procedure based on ISO 14801. The experiments were conducted in air, at a crosshead speed of 1 mm/min (fast enough to avoid slow crack growth during loading), until fracture of the samples. The fracture load was then determined as the maximum of the load-displacement curve.

### 2.4. Scratch Tests

Three zirconia cylinders machined in the same conditions as zirconia implants were used for a scratch test. The samples were aged similarly to the ones used for fracture load measurement (one sample each). Scratch tests were conducted in a self-built scratch tester. Briefly, an alumina pin with a spherical surface was pressed on the top generatrix of the cylindrical samples and dragged along this generator at a fixed speed (15 mm·s^−1^) and with a constant normal load (10 mN). The tangential load necessary to maintain the desired speed was measured, then the friction coefficient was deduced as the ratio between tangential and normal load. This experiment was repeated at least three times on each sample, each time rotating the sample to test a different generatrix.

## 3. Results

### 3.1. Microstructural Analysis

**Figure 1:**
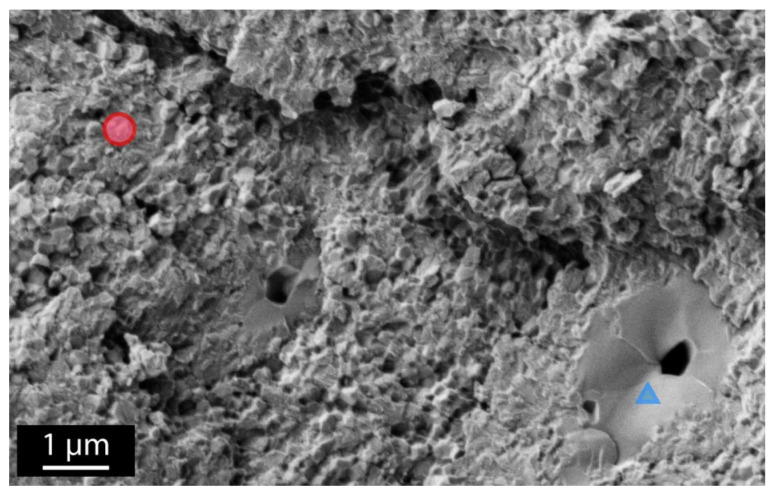
Typical microstructure of the implants. The red circle points to a small-grained zone; the blue triangle indicates a large grain.

All samples exhibited similar microstructures, as illustrated in Figure 1: a mix of small grains with near-nominal yttria content (~2.1 mol.% Y_2_O_3_) and of larger grains containing more yttria (~4.7 mol.%) and often porous in the middle. These big grains are most likely cubic [16], although, given the geometry of the samples, it cannot be checked by XRD in the Bragg–Brentano configuration (the presence of threads involves significant peaks broadening that mask the cubic reflexes).

### 3.2. Ageing Kinetics: Extrapolations to Body Temperature

**Figure 2:**
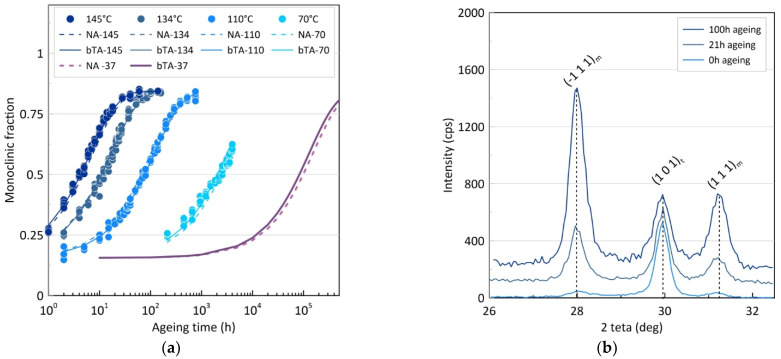
(**a**) Monoclinic volume fraction of dental implants versus ageing time at different temperatures. (**b**) Typical XRD diagrams used for the determination of the monoclinic fraction, shown at three ageing times (0, 21, and 100 h) (for clarity, the diagrams were normalized to near-equal heights of the (101) tetragonal peak).

Figure 2 shows the experimental results of ageing tests at *T* = 70, 95, 110, 134, and 145 °C for different ageing times. As expected, the amount of monoclinic phase increases with both time and temperature. The curves were fitted using the MAJ Equation (Equations (1) and (2)) with a monoclinic content ranging from 17 to 84 volume percentage. Two fitting methods were applied. The first one (numerical analysis, NA) consisted of adjusting five parameters simultaneously—*n*, *b*_0_, *Q*, *f_min_,* and *f_max_*—by fitting all the kinetics simultaneously (using the Generalized Reduced Gradient method implemented in Microsoft Excel solver) while setting an equal weight to each temperature in the error minimization process [17]. The second one (*b-T* analysis—bTA) is the analysis usually applied [11]. It consists of first determining *f_min_* and *f_max_* (from the initial and saturation values of *f*), then *n* for each kinetic (using the slope of the $ln\left(ln\left(\frac{f_{max}-f_{min}}{f_{max}-f}\right)\right)$ versus *ln(t)* diagram). Then, a common value of *n* was decided (the average value for all kinetics) and *b* was calculated for each kinetic (from the intercept of the $ln\left(ln\left(\frac{f_{max}-f_{min}}{f_{max}-f}\right)\right)$ versus *ln(t)* diagrams, imposing the chosen value of *n*). Finally, plotting *ln(b)* versus (*1/T*) gave access to both *b*_0_ and *Q*. The values of the five parameters found using these two adjustment methods are reported in Table 1. This table shows no significant difference between the two sets of parameters. The experimental points and fits are shown in Figure 2. The same figure also shows the ageing kinetics extrapolated to 37 °C, using the two sets of parameters (bTA-37 and NA37).

Given the small differences between the different time–temperature equivalence obtained, it is reasonable to consider that the activation energy measured here is the average of all the ones obtained by the different fitting procedures. Thus, a value of 98.1 kJ·mol^−1^ was selected for Q. This leads to an equivalence of 1 h at 134 °C for 0.99 years at 37 °C, or 1 year at 37 °C for 1.06 h at 134 °C. Therefore, one can roughly state that, for this particular zirconia material, processed under these particular conditions, one hour of hydrothermal ageing under water vapor at 134 °C gives the same monoclinic fraction as one year in vivo at 37 °C.

### 3.3. Mechanical Properties

#### 3.3.1. Load to Fracture

*Sample preparation:* The samples were aged according to the Mehl–Avrami–Johnson Equation determined using the data on ageing kinetics available at the time of the sample preparation (a few points at long times were still missing), to ageing times corresponding to 22.5 years, 45 years, and 90 years in vivo. The selected ageing times are presented in Table 2. Ageing was performed at 134 °C according to the protocol presented in Section 3.2. The monoclinic fraction values are coherent with what was expected from the measured ageing kinetics (although the samples have different geometries and come from different batches).

*Raw results:* Results for load to fracture of both zirconia and titanium implants (average value and standard deviation) are summarized in Table 3. As can be seen, it seems ageing does not have an effect on the value of load to fracture, as the average value of zirconia implants at different ageing times is similar. The standard deviation is also low. Ageing, in the conditions used here, neither reduces nor increases the implants’ strength. However, results for titanium implants are not surprising, with a very large variability (standard deviation four to six times larger than for zirconia implants) and a 40% larger load to fracture.

*Weibull and statistical analyses:* An analysis of variance showed no statistically significant difference between the fracture loads of the implants aged at different times (*p* > 0.05). However, the fracture load of Ti implants was significantly higher than for all other materials (*p* < 0.05).

Fracture loads were also analyzed using a Weibull distribution:

(6) $$p_{f}=1-\exp\left(-{\left(\frac{F}{F_{0}}\right)}^{m}\right)$$

in which *p_f_* is the fracture probability at load *F*, *m* is the Weibull modulus, and *F*_0_ is a normalization factor (the fracture probability is 63% when applying a load equal to *F*_0_*)*.

**Figure 3:**
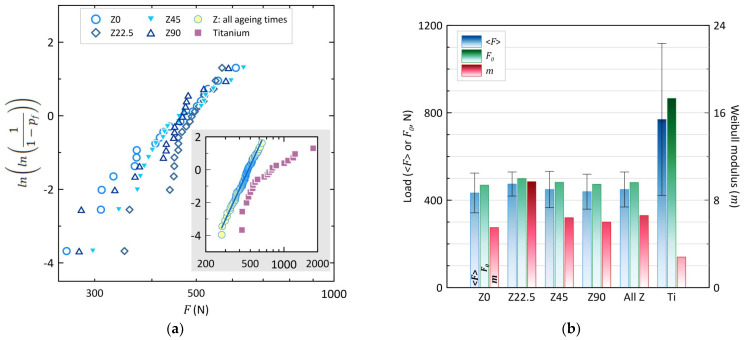
Weibull analysis of the fracture load of zirconia implants. (**a**) Weibull plots for all implants; the main graph shows the individual Weibull plots for zirconia implants with different ageing times; the inset (with same axes as the main graph) shows the Weibull plot for all zirconia implants together and for titanium implants. (**b**) Average fracture loads (<*F*>), central Weibull value (*F*_0_) and Weibull moduli (*m*) on all implants (“All Z”: parameters calculated on all zirconia implants together, regardless of the ageing time).

Figure 3 and Table 3 show the results of this analysis, with *m* values close to 6, indicating a rather distributed load to fracture, which is expected from non-polished samples.

#### 3.3.2. Fractography

**Figure 4:**
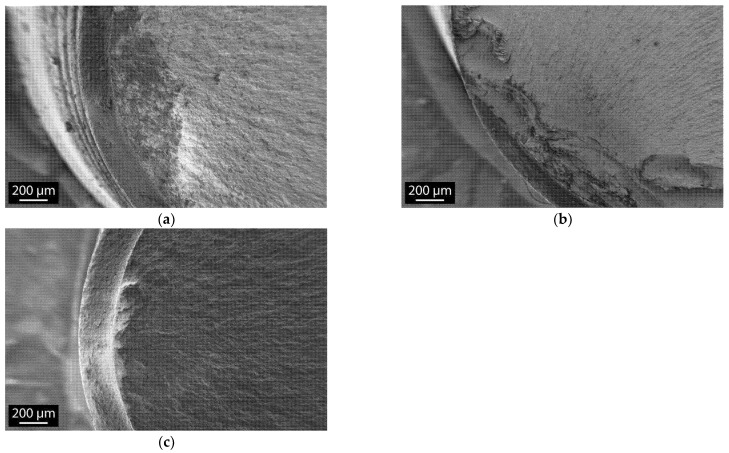
Assumed fracture origin of implants: (**a**) not aged; (**b**) after simulated 45-year ageing; (**c**) after 90-year simulated ageing.

Implants aged at different times were observed in the SEM after fracture. In all cases, the fracture origin was detected on the edge of the fracture surface (Figure 4), indicating a fracture on surface features rather than intrinsic defects. This is true for all samples, whatever their ageing duration. Thus, ageing does not create additional defects able to impact resistance to fracture, in coherence with the observed monodisperse distribution of fracture loads for whatever ageing duration, as observed in the previous section.

#### 3.3.3. Scratch Tests 

**Figure 5:**
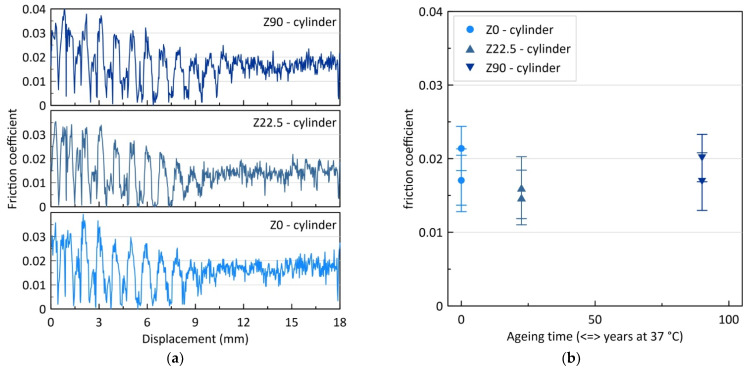
(**a**) Examples of scratch tests results: friction coefficient versus displacement. (**b**) Evolution of friction coefficient with ageing time. Error bars are one standard deviation.

During the scratch test, both the normal (applied) load and the tangential (resulting) load were measured. From these data, the friction coefficient was calculated as the ratio of tangential divided by normal loads. Figure 5a shows the evolution of the friction coefficient with displacement of the pin along the sample. Two stages can be distinguished. In the first stage, large oscillations occur, with approximately the same wavelength for all samples. This is probably due to resonance phenomena in the machine or at the contact, although this hypothesis cannot be proven here. In the second stage, the friction coefficient remains constant. Figure 5b shows the evolution of the friction coefficient measured at stage 2 (for displacement larger than 13 mm) with ageing time. Given the large error bars, no significant evolution could be distinguished. Moreover, micrographs of the scratches did not show any differences between the samples, whatever their ageing time. It can be concluded that, in the condition used here, the scratch test does not allow distinguishing different behaviors between the different surfaces; they all present the same resistance to friction. As a consequence, one can infer that hydrothermal ageing, conducted under these conditions, and on this type of implant, does not degrade the surface in a measurable way.

## 4. Discussion

The microstructure shows a bimodal grain-size distribution, with few large grains in a matrix of fine tetragonal grains. This is most likely due to a partitioning of yttrium during sintering (following the yttria–zirconia phase diagram), which gave rise to yttrium-rich cubic grains (large grains) and (slightly) yttrium-depleted tetragonal grains. Although this depletion of yttrium of the tetragonal phase can be expected to be detrimental to ageing (tetragonal phase being less stable), this was not observed here.

On the contrary, the potentially lesser stability may explain the good mechanical properties obtained on zirconia PURE ceramic implants. For comparison of the strength of the implants analyzed here with other available results on similar implants (from competitors or from previous results on the same implants), it is necessary to take into account the geometry of the implants, particularly their diameters. Assuming that, during mechanical tests, the implants behave like cylinders submitted to flexion, a rough approximation of their strength ($\sigma_{R}$) can be determined from their load to fracture (*F*) by Equation (7):

(7) σR=−16Lsinπ6πFD3

In this equation, *D* is the nominal diameter of the implant, *L* the solicited length (11 mm, to respect ISO 14801), and π6 is the angle between the implant axis and the loading direction (also chosen in agreement with ISO 14801). This value of “approximate strength” is thus shown on the sixth column of Table 3 for different implants. As in first approximation it is independent from the geometry of the implants, it should reflect only the quality of the material and of the machining.

Even though this comparison is limited (for example, it does not account for the depth and shape of the threads), it shows that the 3.3 mm diameter zirconia implants from Straumann have a similar strength to the 4.1 mm diameter ones from the same manufacturer. The results on zirconia PURE ceramic implants obtained in this study are very similar to the ones obtained internally by Straumann on other batches of implants [18], which is a good sign for the reproducibility of the material and of the machining procedure.

The influence of ageing on the mechanical properties is not obvious. The ANOVA did not show any statistically significant difference between the loads to fracture of the zirconia implants aged at different temperatures. Even though the Weibull modulus seems higher at 22.5 years ageing, the significance of this difference is doubtful given the small number of samples (20 per ageing time). In addition, plotting all zirconia samples together in a single Weibull plot shows a monomodal distribution (m = 6.6). Thus, all analyses point to the conclusion that up to 100 years (or rather, 89.9 years—see Table 2) hydrothermal ageing does not influence the fracture load of these dental implants. The same was observed for the friction coefficient, which remains the same whatever the ageing time.

In Y-TZP materials, the role of yttria is to prevent the spontaneous tetragonal-to-monoclinic phase transition by stabilizing the tetragonal phase. However, in the presence of water, hydrothermal ageing counteracts this stabilization. Several possible mechanisms have been identified. In the opinion of the authors, the most probable one is the occupation of oxygen vacancies by hydroxyl ions, which destabilizes the tetragonal phase and overstabilizes the monoclinic one, therefore leading to the t–m phase transformation [19]. It is important to keep in mind, however, that whatever its mechanism, the tetragonal-to-monoclinic phase transformation in the presence of water always progresses from the surface exposed to water to the bulk, being controlled by the diffusion of water and hydroxyl ions into the material. The transformed layer may be seen as a defect that could negatively impact the strength of aged specimens.

However, the tetragonal-to-monoclinic phase transformation results in a large volume increase (5–7%), which imposes compressive stresses to the near-surface [3,4,5,6,7]. These compressive stresses should strengthen the material.

As a result, the evolution of strength with hydrothermal ageing results from a competition between the depth of the transformed zone (usually rather small [20]) and the presence of compressive stresses. Indeed, some studies demonstrate that an increase in strength with ageing time is possible [5]. Here, no effect of the transformation on strength can be seen. The probable explanation is that an equilibrium was reached between the effects of transformation depth and compressive stresses.

## 5. Conclusions

This study aimed at providing an independent characterization of the ageing kinetics of Straumann’s zirconia PURE ceramic implants and of its effect on the mechanical properties.

It showed that:

- The activation energy of ageing is around 98.7 kJ·mol^−1^, meaning that 1 h ageing at 134 °C roughly corresponds to 1 year ageing in vivo.
- Ageing has no measurable effect on the mechanical properties so far: fracture loads are not affected by ageing, and, in the first approximation, scratch tests yield similar results for aged and non-aged samples.
- The microstructure contains big (probably cubic) grains that are enriched in yttria, and smaller grains (probably tetragonal) that contain less yttria. This partitioning of yttria may explain both the good mechanical properties and the sensitivity to ageing.

A more careful microstructural analysis of the near surface after ageing, exploring the microcracks network in particular (if there is any), would complete the study to ensure that the aged surfaces remain strongly attached to the samples.

## Figures and Tables

**Table 1:** Constants of the MAJ Equation calculated from the fitted curves, considering all temperatures.

Fit Type	NA	bTA	Average
*n*	0.8	0.8	
*Q* (kJ mol^−1^)	99.1	97.1	98.1
*b*_0_ (h^−1^)	3.14 × 10^11^	1.71 × 10^11^	
*f_min_* (%)	15.5	15.5	15.5
*f_max_* (%)	84.3	84.3	84.3
1 h at 134 °C is X years at 37 °C	1.08	0.89	0.99
1 year at 37 °C is Y hours at 134 °C	0.92	1.11	1.01

**Table 2:** Ageing time and monoclinic phase content of zirconia implants. Third column is calculated using a 98.1 kJ·mol^−1^ activation energy.

Condition	Ageing Time at 134 °C	Real Equivalent Ageing Time at 37 °C (Years)	Monoclinic Vol. Fraction (%)
Z0	0	0	11
Z22.5	22 h 46 min	22.5	65
Z45	45 h 33 min	45	77
Z90	91 h 6 min	89.9	82

**Table 3:** Results for load to fracture for zirconia and titanium implants (four last lines: results available in the literature for other one-piece implants); <*F*>: average load to fracture (N) (given ± one standard deviation); *σ_R_*: approximate strength (MPa) deduced from Equation (7); *F*_0_ and *m*: parameters of the Weibull distribution (Equation (6)).

Ref.	Condition/Material/Geometry	<*F*> (N) ± 1 Std Dev	*F*_0_	*m*	$\sigma_{R}$
This study	Z0 (d = 3.3 mm)	433 ± 91	469	5.5	338
	Z22.5 (d = 3.3 mm)	474 ± 55	499	9.7	369
	Z45 (d = 3.3 mm)	449 ± 83	482	6.4	350
	Z90 (d = 3.3 mm)	439 ± 80	473	6.0	342
	Zirconia (d = 3.3 mm), all ageing times	449 ± 80	481	6.6	350
	Titanium	770 ± 348	866	2.8	600
[18]	Straumann PURE ceramic implants (d = 4.1 mm)	730			297
[18]	Straumann PURE ceramic implants (d = 3.3 mm)	423			330
[18]	Z-system Z-look Evo rapide implants (d = 4 mm)	489			214
[18]	Z-system Z-look Evo rapide implants (d = 3.6 mm)	368			221

## Data Availability

Data are available upon request to the corresponding autor.
